# Erfolgreiche Behandlung eines Lupus erythematodes tumidus (LET) mit Deucravacitinib bei einer Patientin mit gleichzeitig vorliegender Plaquepsoriasis

**DOI:** 10.1007/s00105-026-05713-2

**Published:** 2026-06-02

**Authors:** Tim Schricker, Antonia Matiszick, Ulrike Raap, Nikolaos Patsinakidis

**Affiliations:** 1 Universitätsklinik für Dermatologie und Allergologie, Klinikum Oldenburg AöR, Rahel-Straus-Str. 10, 26133 Oldenburg, Deutschland; 2 Abteilung für experimentelle Allergologie und Immundermatologie, Carl von Ossietzky Universität Oldenburg, Oldenburg, Deutschland

**Keywords:** Intermittierend kutaner Lupus erythematodes (ICLE), Therapiealternativen, TYK2-Inhibition, Düsseldorfer Klassifikation, Kollagenosen, Intermittent cutaneous lupus erythematosus (ICLE), Treatment options, TYK2 inhibitor, Düsseldorf classification, Collagenosis

## Abstract

Wir berichten über eine 48-jährige Patientin mit mittelschwerer Plaquepsoriasis und Lupus erythematodes tumidus (LET), die unter Deucravacitinib 6 mg/Tag eine rasche Besserung des LET sowie eine deutliche Verbesserung der Plaquepsoriasis zeigte. Die Kasuistik verdeutlicht die potenzielle Wirksamkeit des Tyrosinkinase 2(TYK2)-Inhibitors Deucravacitinib bei Patienten mit Lupus erythematodes tumidus.

## Abstract (en)

We present a 48-year-old woman with moderate refractory plaque psoriasis and lupus erythematosus tumidus (LET) who experienced rapid LET improvement and significant psoriasis response under deucravacitinib 6 mg/day. This case highlights the possible role of tyrosine kinase 2 (TYK2) inhibition with deucravacitinib in the treatment of LET.

## Anamnese

Eine 48-jährige Patientin stellte sich mit einer langjährigen mittelschweren Plaquepsoriasis vor. Vortherapien umfassten topische Steroide, Phototherapien und Fumarsäureester. Unter Methotrexat (MTX) 17,5 mg s.c. wöchentlich zeigte sich weiterhin keine ausreichende Krankheitskontrolle. Zusätzlich bestanden seit mehreren Jahren chronische, rezidivierende und teils UV(Ultraviolett)-getriggerte livid-erythematöse Plaques ohne Schuppung, histologisch und klinisch vereinbar mit einem Lupus erythematodes tumidus (LET). In der serologischen Diagnostik waren keine antinukleären Antikörper (ANA) nachweisbar. Eine Therapie des LET mit Hydroxychloroquin hatte zuvor zu einer Verschlechterung der Plaquepsoriasis geführt. An Vorerkrankungen bestehen eine depressive Störung, begleitet von Angst- und Panikattacken, sowie eine chronische Schmerzstörung und langjähriger Nikotinabusus.

## Befund

**Abb. 1:**
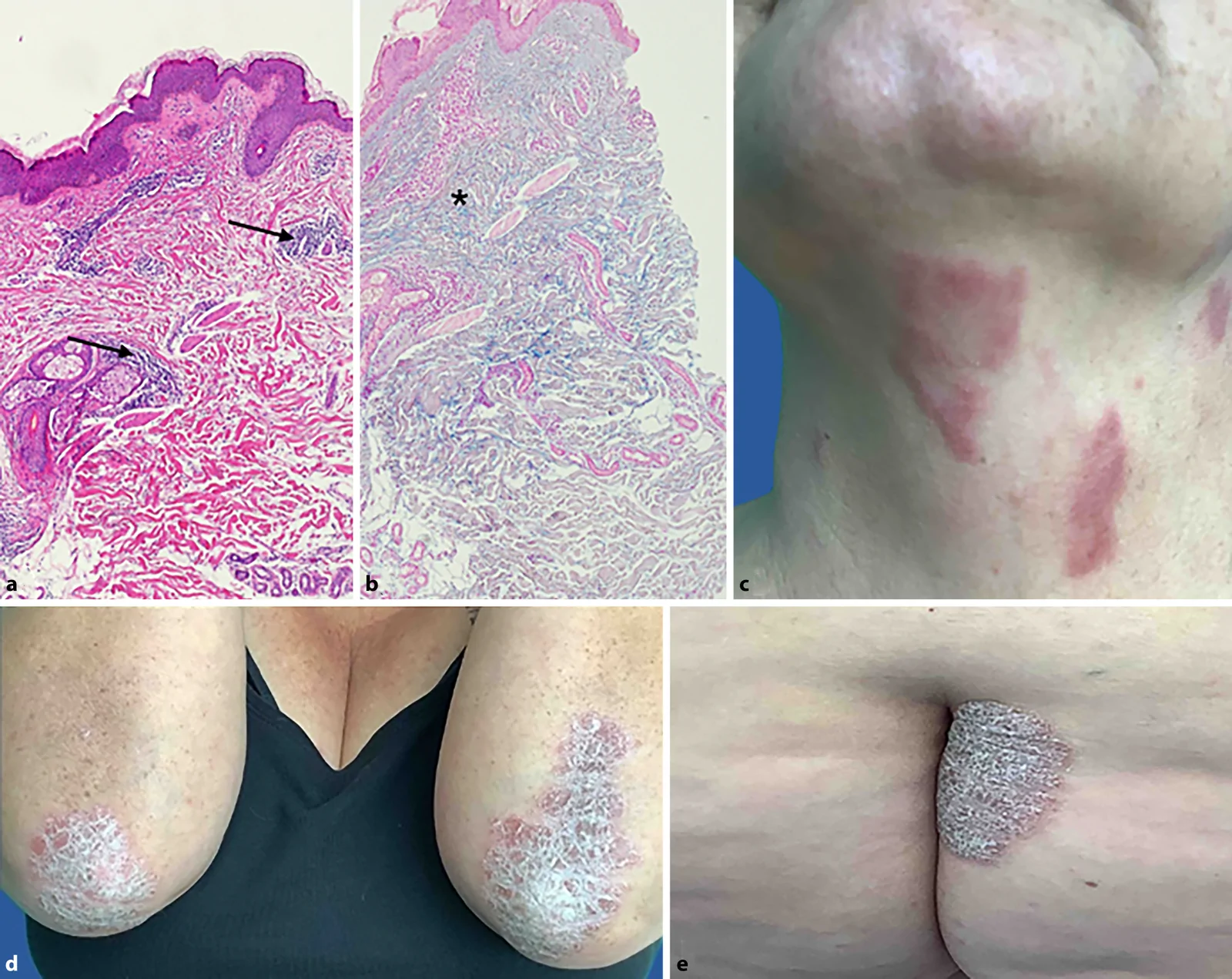
Histologische und klinische Befunde. **a** Hämatoxylin-Eosin(H&E)-Färbung mit periadnexiellen (*erster Pfeil*) und perivaskulären (*zweiter Pfeil*) lymphozytären Infiltraten in der Dermis unter 20-facher Vergrößerung. **b** Alcianblau-Färbung mit interstitiellen Muzinablagerungen in der Dermis zwischen den Kollagenfaserbündeln (*Sternchen*) unter 20-facher Vergrößerung. **c–e** Hautveränderungen vor Umstellung der Therapie

Klinisch bestanden im vorderen Halsbereich sowie am linken Schulterblatt scharf begrenzte glatte livid-erythematöse urtikarielle Plaques ohne epidermale Beteiligung, vereinbar mit dem vorbekanntem Lupus erythematodes tumidus (Cutaneous lupus erythematosus disease area and severity index(CLASI)-Score 6). Zusätzlich zeigten sich am Rumpf und an den Streckseiten der Extremitäten scharf begrenzte erythematöse Plaques mit fest anhaftender silbrig-weißer Schuppung, vereinbar mit der Diagnose der Plaquepsoriasis (Psoriasis area and severity index(PASI)-Score 7,5) (Abb. 1). Das histologische Ergebnis einer Stanzbiopsie zervikal zeigte ein periadnexielles und perivaskuläres lymphozytäres dermales Infiltrat neben interstitiellen Muzinablagerungen (Abb. 1a, b). Ein sehr starker Einfluss auf die Lebensqualität lag vor (DLQI-Score 22).

## Therapie und Verlauf

Bei der Patientin lagen eine therapierefraktäre Plaquepsoriasis sowie ein LET vor. Wir beendeten die Therapie mit MTX und begannen eine Therapie mit Deucravacitinib.

**Abb. 2:**
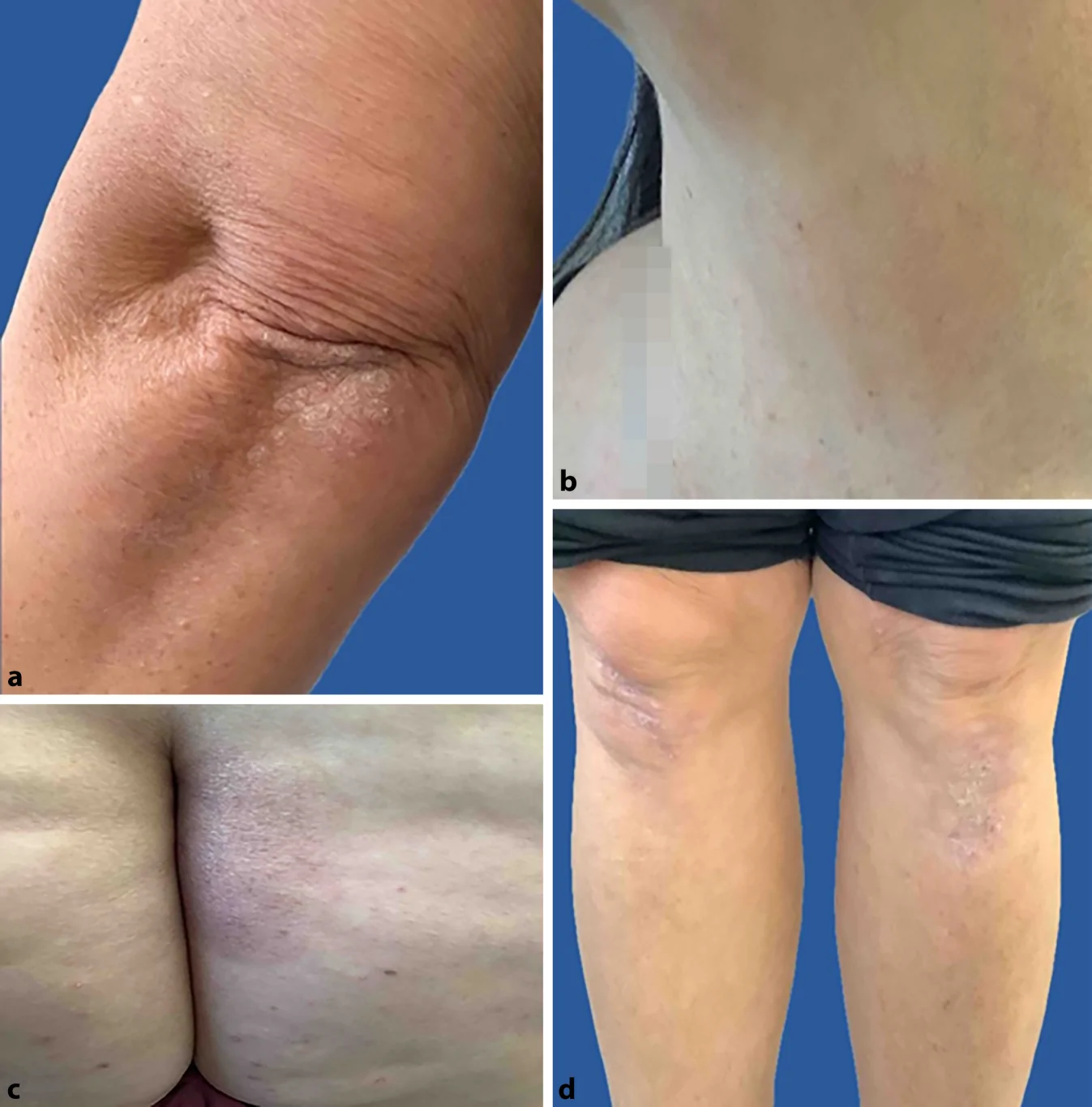
Hautveränderungen 5 Monate nach Umstellung auf Deucravacitinib. **a** Ellenbogenstreckseiten. **b** vorderer Halsbereich. **c** gluteal. **d** Kniegelenksstreckseiten

Bereits nach einem Monat zeigte sich unter Deucravacitinib (6 mg/Tag p.o.) eine schnelle und deutliche Rückbildung der LET-Läsionen bei guter Verträglichkeit. Gleichzeitig berichtete die Patientin über eine subjektive Abnahme des Brennens und des Spannungsgefühls der Haut. Nach 5 Monaten zeigten sich sowohl der LET als auch die Plaquepsoriasis in einem nahezu erscheinungsfreien Zustand (Abb. 2). Relevante Nebenwirkungen traten nicht auf. Ein positiver Einfluss auf das bestehende Schmerzsyndrom wurde unter der Therapie nicht beobachtet.

## Diskussion

Die systemische Therapie des CLE stellt weiterhin eine Herausforderung dar, insbesondere für Patient:innen die auf Antimalariamittel (z. B. Hydroxychloroquin) entweder nicht ansprechen oder sie nicht vertragen haben [1]. Moderne zielgerichtete Therapiestrategien rücken daher zunehmend in den Fokus. Deucravacitinib ist ein TYK2-Inhibitor, der gute Wirksamkeit und Verträglichkeit in der Behandlung der mittelschweren bis schweren Psoriasis aufgewiesen hat [2], aber auch das Potenzial für Off-label-Anwendungen zeigt [3]. Durch die TYK2-Inhibition werden nicht nur die psoriatische Entzündungsachse (Th17-gesteuert), sondern auch die für den Lupus relevanten Typ-I-Interferon-Signalwege inhibiert [4]. In einer aktuellen Arbeit zeigte eine Inhibition von TYK2 nicht nur eine klinische Wirksamkeit, sondern sie normalisierte auch die in-vitro krankheitsrelevanten molekularen Signaturen in unterschiedlichen Subtypen des kutanen Lupus erythematodes [5]. Des Weiteren besteht eine mit der Zeit zunehmende Evidenz über die erfolgreiche Therapie von Patient:innen mit kutanem Lupus erythematodes (CLE) mit Deucravacitinib. Eine aktuelle systematische Metaanalyse von Bokor et al. untersuchte die Wirksamkeit moderner therapeutischer Ansätze bei CLE unter besonderer Berücksichtigung des CLASI-50-Ansprechens [6]. Die Arbeit zeigte, dass unter Deucravacitinib 8‑mal mehr Patienten ein CLASI-50-Ansprechen erreichten als unter Placebo, was die bislang stärkste dokumentierte Effektgröße für eine Therapie in diesem Indikationsgebiet widerspiegelt. Auch die Biologika Litifilimab („anti-blood dendritic cell antigen 2“ [BDCA-2]; hemmt die Produktion von Typ-I-Interferonen) und Anifrolumab (Anti-Interferon-α/β-Rezeptor 1 [IFNAR1]; blockiert die Signalübertragung aller Typ-I-Interferone) zeigten in dieser Analyse eine mehr als doppelte Wahrscheinlichkeit für das Erreichen eines CLASI-50-Scores zu Placebo bei gleichzeitig gutem Sicherheitsprofil [6], was das therapeutische Potenzial solcher Medikamente in der Behandlung des CLE nochmals unterstreicht. Neben den Ergebnissen kontrollierter Studien nimmt auch die Zahl publizierter Fallberichte zu, die einen erfolgreichen Einsatz von Deucravacitinib bei verschiedenen Subtypen des CLE beschreiben. Mittlerweile gibt es 9 klinische Fallberichte, die einen positiven Effekt von Deucravacitinib für den subakuten kutanen Lupus erythematodes (SCLE), diskoiden Lupus erythematodes (DLE), Lupus erythematodes tumidus (LET) und Chilblain Lupus (ChLE) aufzeigen [7]. Diese zunehmende Fallzahl deutet darauf hin, dass die selektive Hemmung von TYK2 ein therapeutisches Fenster eröffnet, das insbesondere bei komplexen oder therapieresistenten dermatologischen Krankheitsbildern relevant sein kann. Deucravacitinib ist derzeit nicht für die Behandlung des CLE zugelassen. Das rasche Ansprechen der LET-Läsionen innerhalb von 4 Wochen stützt die Evidenz für einen potenziellen Nutzen bei komplexen CLE-Verläufen. Aufgrund der begrenzten klinischen Datenlage sind jedoch weitere Studien erforderlich.

## Fazit für die Praxis

- Aktuelle Studien- und Fallberichte weisen bis dato auf einen relevanten klinischen Nutzen von Deucravacitinib bei kutanem Lupus erythematodes hin.
- Wir beschreiben den Fall einer 48-jährigen Patientin mit Lupus erythematodes tumidus und mittelschwerer Plaquepsoriasis, die unter Deucravacitinib 6 mg/Tag eine rasche Besserung des LET sowie ein deutliches Ansprechen der Plaquepsoriasis zeigte.
- Die Anwendung von Deucravacitinib kann als gut verträgliche und effektive Therapiealternative bei kutanem Lupus erythematodes einschließlich Lupus erythematodes tumidus erwogen werden.

## Data Availability

Alle dieser Arbeit zugrunde liegenden Daten sind in diesem Artikel enthalten.
